# Development of a method for evaluating the mRNA transcription activity of influenza virus RNA-dependent RNA polymerase through real-time reverse transcription polymerase chain reaction

**DOI:** 10.1186/s12985-021-01644-7

**Published:** 2021-08-28

**Authors:** Yuka Horio, Mototada Shichiri, Yuji Isegawa

**Affiliations:** 1 Department of Food Sciences and Nutrition, Mukogawa Women’s University, 6-46 Ikebiraki, Nishinomiya, Hyogo 663-8558 Japan; 2 Institute for Biosciences, Mukogawa Women’s University, 6-46 Ikebiraki, Nishinomiya, Hyogo 663-8558 Japan; 3 Biomedical Research Institute, National Institute of Advanced Industrial Science and Technology (AIST), 1-8-31 Midorigaoka, Ikeda, Osaka 563-8577 Japan; 4 DBT-AIST International Laboratory for Advanced Biomedicine (DAILAB), 1-1-1 Higashi, Tsukuba-shi, Ibaraki, 305-8562 Japan

**Keywords:** Influenza virus, RNA-dependent RNA polymerase activity, Real-time reverse transcription polymerase chain reaction, Ribavirin

## Abstract

**Background:**

The development of an influenza RNA-dependent RNA polymerase (RdRp) inhibitor is required; therefore, a method for evaluating the activity of influenza RdRp needs to be developed. The current method uses an ultracentrifuge to separate viral particles and quantifies RdRp activity with radioisotope-labeled nucleosides, such as ^32^P-GTP. This method requires special equipment and radioisotope management, so it cannot be implemented in all institutions. We have developed a method to evaluate the mRNA transcription activity of RdRp without using ultracentrifugation and radioisotopes.

**Results:**

RdRp was extracted from viral particles that were purified from the culture supernatant using anionic polymer-coated magnetic beads that can concentrate influenza virus particles from the culture supernatant in approximately 30 min. A strand-specific real-time reverse transcription polymerase chain reaction (RT-PCR) method was developed based on reverse transcription using tagged primers. RT primers were designed to bind to a sequence near the 3' end of mRNA containing a poly A tail for specific recognition of the mRNA, with an 18-nucleotide tag attached to the 5' end of the sequence. The RT reaction was performed with this tagged RT primer, and the amount of mRNA was analyzed using real-time qPCR. Real-time qPCR using the tag sequence as the forward primer and a segment-specific reverse primer ensured the specificity for quantifying the mRNA of segments 1, 4, and 5. The temperature, reaction time, and Mg^2+^ concentration were determined to select the optimum conditions for in vitro RNA synthesis by RdRp, and the amount of synthesized mRNAs of segments 1, 4, and 5 was determined with a detection sensitivity of 10 copies/reaction. In addition, mRNA synthesis was inhibited by ribavirin triphosphate, an RdRp inhibitor, thus indicating the usefulness of this evaluation method for screening RdRp inhibitors.

**Conclusion:**

This method makes it possible to analyze the RdRp activity even in a laboratory where ultracentrifugation and radioisotopes cannot be used. This novel method for measuring influenza virus polymerase activity will further promote research to identify compounds that inhibit viral mRNA transcription activity of RdRp.

**Supplementary Information:**

The online version contains supplementary material available at 10.1186/s12985-021-01644-7.

## Background

The influenza virus is an enveloped negative-strand RNA virus, and the viral particles contain eight genomic RNA segments encoding viral proteins [1, 2]. Each RNA segment contains a ribonucleoprotein (RNP) with a heterotrimeric RNA polymerase complex containing the viral acidic polymerase protein (PA), polymerase basic protein 1 (PB1), polymerase basic protein 2 (PB2), and nucleoprotein (NP) [2]. Viral RNPs (vRNPs) are involved in the transcription of viral genes and replication of the viral RNA genome in infected cells. The process of influenza virus replication by RdRp involves three RNAs: mRNA, viral RNA (vRNA), and complementary RNA (cRNA). RdRp first transcribes vRNA into mRNA, which is used to produce viral proteins. This is called primary transcription. Moreover, the replication reaction synthesizes cRNA that is completely complementary to vRNA. RdRp then replicates the new vRNA using cRNA as a template [3]. Recent studies suggest that de novo polymerase complexes are required for the virus to replicate vRNA [4].

Although neuraminidase inhibitors, such as oseltamivir and zanamivir, are one of the mainstream treatments for influenza, an epidemic of neuraminidase inhibitor-resistant viruses has been reported [5]. Baloxavir marboxil, which has been in clinical use since 2018 in Japan, the United States, and other countries, potently and selectively inhibits the cap-dependent endonuclease within the polymerase PA subunit of influenza A and B viruses [6]. However, after treatment with baloxavir marboxil, amino acid substitutions at position 38 in the influenza virus PA (PA/I38T) subunit have occasionally been observed in pediatric and adult patients (23% and 10%, respectively). It has been reported that the susceptibility of the PA/I38T mutant virus to baloxavir marboxil has reduced by approximately 50-fold [7].

T-705 (favipiravir) and ribavirin have been reported as effective influenza virus RNA-dependent RNA polymerase (RdRp) inhibitors in in vitro and in vivo experiments [8, 9]. These compounds are analogs of nucleosides and interrupt RNA synthesis through viral RdRp. Humans do not have RdRp but they do have DNA-dependent RNA polymerase and DNA-dependent DNA polymerase. Animal experiments to assess the teratogenicity of favipiravir using four separate species, mice, rat, rabbits and monkeys, demonstrate delayed development or embryonic death in the first trimester of pregnancy [10]. For this reason, the clinical use of favipiravir in women of reproductive age comes with a strong warning. Therefore, it is necessary to develop an inhibitor that can specifically inhibit influenza RdRp without affecting RNA and DNA synthesis in infected hosts.

A method for evaluating RdRp activity is important to screen for viral RdRp inhibitors. In a previously reported method, the virus is purified through density gradient ultracentrifugation [11–13]. However, this step is time consuming and the purification process is complicated. In addition, a polymerase reaction is performed in vitro using a radioisotope-labeled nucleoside, such as ^32^P-GTP, and the synthesized RNA is determined with a scintillation counter. Even though this method is capable of determining even a very small amount of RNA, the use of a radioisotope is a safety concern.

In this study, we developed a novel method for the evaluation of RdRp activity for screening of RdRp inhibitors that do not use density gradient ultracentrifugation or radioisotopes. Especially, we focused on developing a method for quantifying mRNA transcription activity of RdRp rather than a vRNA replication activity that requires de novo RdRp and a two-step synthesis process in which vRNA is synthesized after cRNA synthesis.

## Materials and methods

### Cell culture

Madin-Darby canine kidney (MDCK) cells were grown in Eagle’s minimum essential medium (MEM; FUJIFILM Wako Pure Chemical Industries, Ltd., Osaka, Japan) containing 7% fetal bovine serum (FBS; Biowest SAS, Nuaillé, France) and 1% antibiotics (penicillin and streptomycin; FUJIFILM Wako).

### Viruses

Influenza virus A/Puerto Rico/8/34 (PR8, H_1_N_1_) was used in this study. For cell infection, the virus was diluted in serum-free MEM supplemented with 0.4 g/L bovine serum albumin (FUJIFILM Wako) and adsorbed to cells at a multiplicity of infection (MOI) of 0.001 for 1 h at 37 °C. The inoculum was then removed and replaced with FBS-free Dulbecco’s modified Eagle medium (DMEM; FUJIFILM Wako) supplemented with 4 g/L bovine serum albumin and acetyltrypsin (2 µg/mL; Sigma-Aldrich, St. Louis, MO, USA) for 24 h.

### Purification of the influenza virus RdRp

Influenza virus RNA polymerase was purified from viral culture supernatants using Viro-Adembeads (Ademtech, Pessac, France) according to the manufacturer’s instructions [14]. This bead is an anionic polymer-coated magnetic beads that can concentrate influenza virus particles from the culture supernatant in approximately 30 min (Additional file 1: Fig. S1). Briefly, 500 μL of a virus solution (4.8 × 10^6^ FFU/mL) was adsorbed onto magnet beads by mixing at 900 rpm for 20 min at 20 °C. The tube was placed on the magnet for 1 min, the supernatant was removed, and the beads were washed with phosphate-buffered saline (PBS). After that, 70 µL of the polymerase elution buffer (50 mM Tris–HCl pH 8.0, 0.1 M KCl, 5 mM MgCl_2_, 2 mM DTT, 1000 U/mL RNase inhibitor, and 0.25% Triton N-101) was added, and the mixture was incubated at 30 °C for 30 min to extract the RdRp.

### In vitro transcription with purified RdRp

The in vitro transcription was conducted using a standard RNA synthesis reaction buffer [9, 12, 13, 15], which contained 50 mM Tris–HCl (pH 8.0), 0.1 M KCl, 5 mM MgCl_2_, 2 mM DTT, 1000 U/mL RNase inhibitor, 0.25% Triton N-101, 500 μM ATP, 500 μM CTP, 500 μM UTP, 500 μM GTP, 200 μM ApG dinucleotide, and 20 µL purified RdRp. The reaction was performed at 37 °C for 30 min in a final volume of 50 µL.

### Evaluation of RdRp activity through real-time reverse transcription polymerase chain reaction (RT-PCR)

RdRp activity was evaluated using RT-PCR. mRNA specific RT primers (Table 1) for segments 1 (PB2), 4 (HA), and 5 (NP) of the PR8 strain were designed by modifying a previously reported protocol [16]. To prepare cDNA from the viral RNA produced through the aforementioned RdRp reaction, each tagged-specific RT primer and the ReverTra Ace qPCR RT Kit (TOYOBO, Osaka, Japan) were mixed as follows: 5 μL of a mixture containing 4 μL of the RdRp reaction product and 1 μL of the 5 pmol tagged-specific RT primer. The mixture was heated at 65 °C for 10 min, immediately cooled on ice for 5 min, and then heated again at 60 °C for 5 min. 5X RT buffer (containing reaction buffer, MgCl_2_, and dNTPs), RT enzyme mix (containing RT enzyme and RNase inhibitor), and nuclease-free water were added to the RdRp product and primer mixture to a total reaction volume of 10 μL. Real-time quantitative PCR (qPCR) was performed on an ABI PRISM 7500 Fast Real-time PCR system using the Thunderbird SYBR qPCR mix (TOYOBO) and the primer sets for each segment mRNA (Table 1). Each 20 µL of the PCR mixture contained 2 µL of a ten-fold dilution of the cDNA and 0.3 µM of forward and reverse primers. The amplification conditions were 95 °C for 1 min, 40 cycles at 95 °C for 15 s, 60 °C for 30 s, and 72 °C for 35 s. Copy numbers were estimated from a calibration curve obtained using serial tenfold dilutions (10^9^, 10^8^, 10^7^, 10^6^, 10^5^, 10^4^, 10^3^, and 10^2^) and 10 copies of the quantified standard sample as the template. Standard samples were created by inserting the respective amplification range sequences into the pEGFP-N1 plasmid.

**Table 1:** Primer sequences for quantitative real-time PCR

Target	Purpose	Prime name	Sequences (5’ to 3’)
Segment 1 mRNA	Reverse transcription	PR8 seg1 mRNA tag	CCAGATCGTTCGAGTCGTTTTTTTTTTTTTTTTTAAACTATTCGA
	Realtime PCR	mRNAtag	CCAGATCGTTCGAGTCGT
		PR8 seg1 mRNA Re	GGAGATATGGGCCAGCATTA
Segment 4 mRNA	Reverse transcription	PR8 seg4 mRNA tag	CCAGATCGTTCGAGTCGTTTTTTTTTTTTTTTTTCCTCATATTTCT
	Realtime PCR	mRNAtag	CCAGATCGTTCGAGTCGT
		PR8 seg4 mRNA Re	GGGCAATCAGTTTCTGGATGTGTTCT
Segment 5 mRNA	Reverse transcription	PR8 seg5 mRNA tag	CCAGATCGTTCGAGTCGTTTTTTTTTTTTTTTTTCTTTAATTGTC
	Realtime PCR	mRNAtag	CCAGATCGTTCGAGTCGT
		PR8 seg5 mRNA Re	CGATCGTGCCTTCCTTTG
Target	Purpose	Prime name	Sequences (5’ to 3’)
Segment 1 mRNA	Reverse transcription	PR8 seg1 mRNA tag	CCAGATCGTTCGAGTCGTTTTTTTTTTTTTTTTTAAACTATTCGA
	Realtime PCR	mRNAtag	CCAGATCGTTCGAGTCGT
		PR8 seg1 mRNA Re	GGAGATATGGGCCAGCATTA
Segment 4 mRNA	Reverse transcription	PR8 seg4 mRNA tag	CCAGATCGTTCGAGTCGTTTTTTTTTTTTTTTTTCCTCATATTTCT
	Realtime PCR	mRNAtag	CCAGATCGTTCGAGTCGT
		PR8 seg4 mRNA Re	GGGCAATCAGTTTCTGGATGTGTTCT
Segment 5 mRNA	Reverse transcription	PR8 seg5 mRNA tag	CCAGATCGTTCGAGTCGTTTTTTTTTTTTTTTTTCTTTAATTGTC
	Realtime PCR	mRNAtag	CCAGATCGTTCGAGTCGT
		PR8 seg5 mRNA Re	CGATCGTGCCTTCCTTTG

## Results

### Primer design and standard curve for mRNA segments 1, 4, and 5

To quantify the copy numbers of mRNA of segments 1, 4, and 5 in the PR8 strain, we designed primers specific for each segment (Table 1) by modifying the primer sequences previously reported by Kawakami et al.[16]. First, tagged primers for cDNA synthesis were used as previously reported [16]. This 18-nucleotide tag (mRNAtag; CCAGATCGTTCGAGTCGT) was not related to the influenza virus and attached to the 5’ end of the primer for RT reaction (Table 1 and Additional file 1: Fig. S1). Influenza virus vRNA is a negative-sense single-stranded RNA, whereas the cRNA and mRNA are positive-sense single-stranded RNAs. As the cRNA and mRNA sequences and the vRNA sequence are complementary, it is possible to distinguish vRNA from the other two RNAs using appropriate RT primers. The cRNA and mRNA sequences differ in the cap structure at the 5' end and the poly A tail at the 3' end. In order to specifically bind to mRNA, a sequence with a tag sequence attached to the sequence near the 3' end of mRNA, including the poly A tail, was used as the RT primer. In real-time qPCR, the tag sequence was used as the forward primer, and the reverse primer was selected as a sequence that does not bind to vRNA, cRNA, or mRNA of other segments (Table 1).

**Fig. 1:**
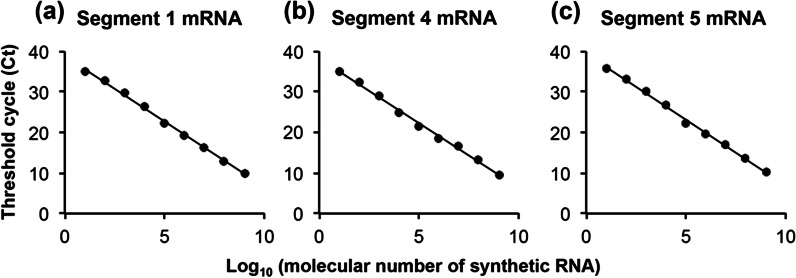
Standard curve for segments 1 (**a**), 4 (**b**), and 5 (**c**). A standard curve was generated by plotting the threshold cycle (Ct). Ten-fold serial dilution (10^1^–10^9^ copies/µL) of standard DNA were used to generate a standard curve

Calibration curves for each segment mRNA were constructed (Fig. 1a–c). Each calibration curve was linear in the range of 10^1^–10^9^ copies in the reaction solution. The linear correlations of the calibration curves in this range were r = 0.999 in segment 1, r = 0.998 in segment 4, and r = 0.999 in segment 5 (Fig. 1a–c, Table 2).

**Table 2:** Validation parameters for mRNA quantification of segments 1, 4, and 5 by strand-specific real-time RT-PCR using tagged primers

Target	Sensitivity (copies)					Linear regression
		Slope	Intercept	Amplification efficiency (E%)	R^2^	
Segment 1	mRNA	10^1^	− 3.2232	38.833	100	0.9979
Segment 4	mRNA	10^1^	− 3.1873	38.24	100	0.9967
Segment 5	mRNA	10^1^	− 3.2491	39.379	100	0.9972

### Effect of the polymerase reaction time on influenza virus mRNA synthesis

Presence of the proteins PA, PB1, PB2, and NP, components of RdRp, in the extract solution of RdRp was confirmed by western blotting (Additional file 1: Fig. S2). In addition, viruses purified using these beads have been reported to have infectious activity [14]. In addition, the solvent used for extracting RdRp from the bead-purified virus is the same solvent as previously reported for analyzing the RdRp activity of influenza virus [11–13]. From these results and those obtained from a previous study, RdRp could be extracted successfully in a form that retains its enzymatic activity.

**Fig. 2:**
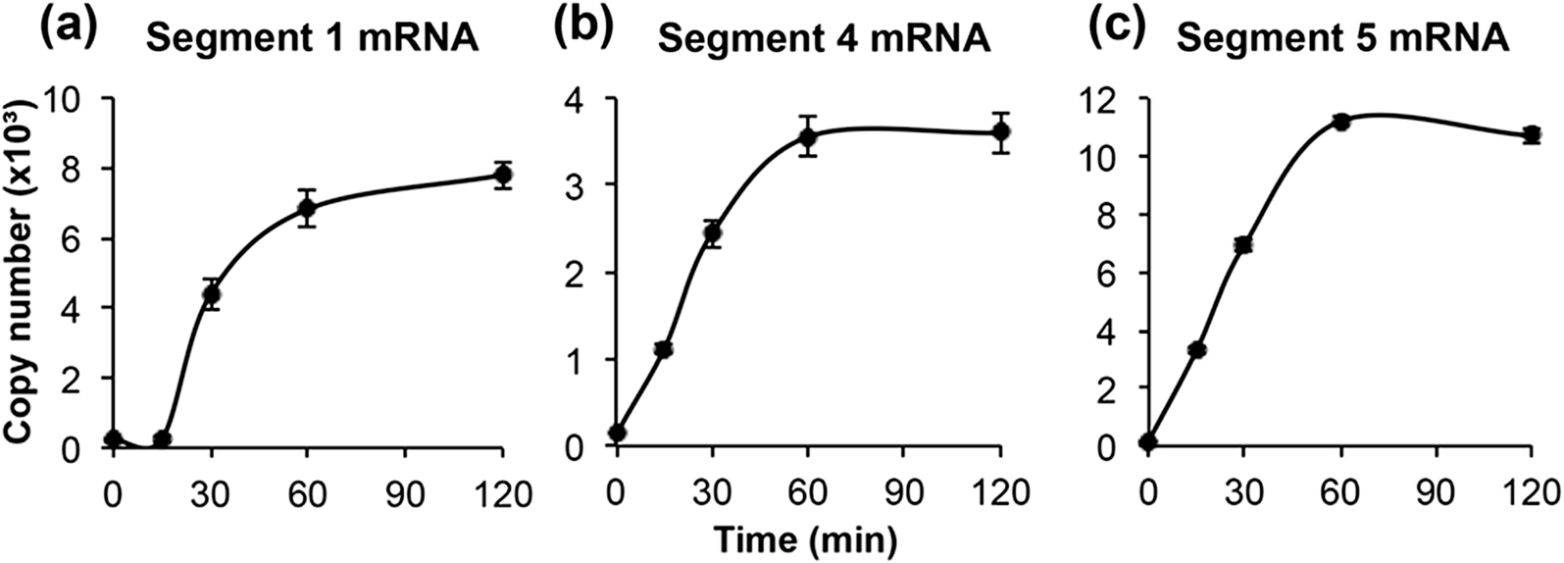
Effect of the reaction time on the influenza virus mRNA synthesis. The RNA polymerase reaction mixture containing 5 mM of MgCl_2_ was incubated for 0, 15, 30, 60, and 120 min at 37 °C. **a** Segment 1 mRNA, **b** segment 4 mRNA, and **c** segment 5 mRNA. Data are presented as mean ± standard deviation (n = 3)

The optimum conditions for the RdRp reaction were examined. After the RdRp reaction for 0, 15, 30, 60, and 120 min, mRNA production was maximal at 120 min in segment 1 (7.8 ± 0.39 × 10^3^ copies in reaction), maximal at 60 min in segment 4 (3.6 ± 0.23 × 10^3^ copies in reaction), and maximum at 60 min in segment 5 (1.1 ± 0.01 × 10^4^ copies in reaction) (Fig. 2a–c). Therefore, the RdRp reaction time was 30 min that corresponds to half of the maximum reaction rate for each segment.

### Influenza virus mRNA synthesis at various temperatures

**Fig. 3:**
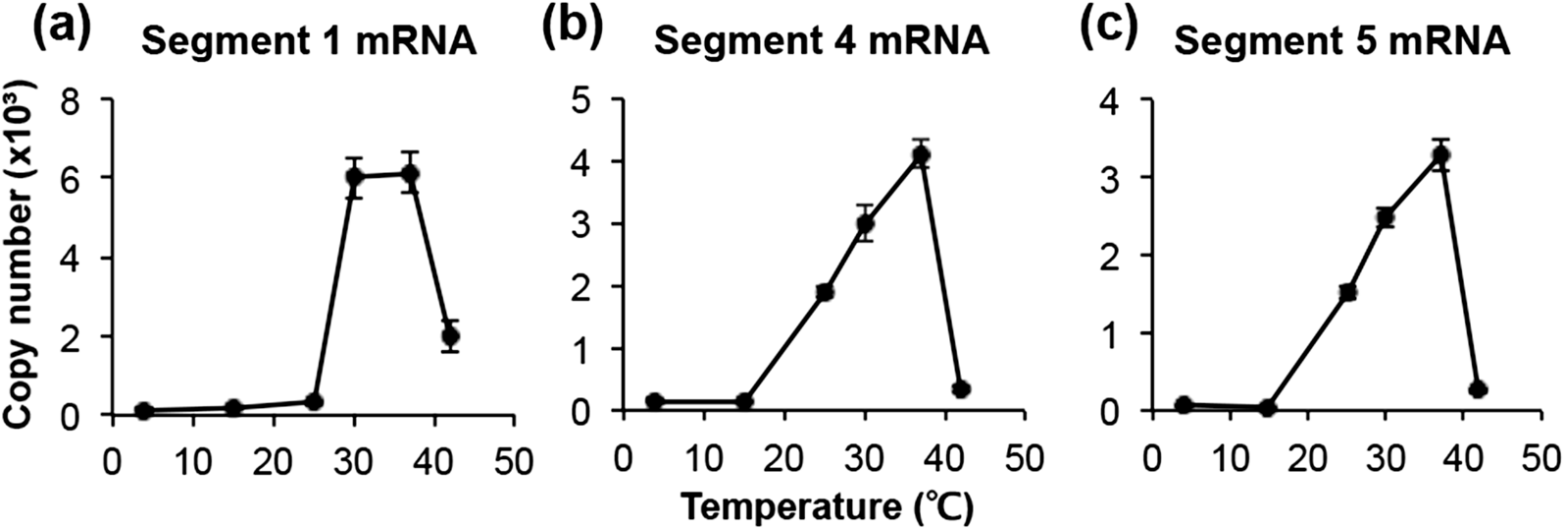
Influenza virus mRNA synthesis at various temperatures. The RNA polymerase reaction mixture containing 5 mM of MgCl_2_ was incubated at 4, 15, 25, 30, 37, and 42 °C for 30 min. **a** Segment 1 mRNA, **b** segment 4 mRNA, and **c** segment 5 mRNA. Data are presented as mean ± standard deviation (n = 3)

The optimum temperature for the RdRp reaction was examined in the range of 4–42 °C. The highest activity was observed in segment 1 at 30–37 °C (Fig. 3a), and in segments 4 and 5, mRNA production increased in a temperature-dependent manner between 15 °C and 37 °C (Fig. 3b, c). In all segments, RdRp activity decreased under high temperature conditions of 42 °C (Fig. 3a–c). Therefore, the reaction temperature of RdRp was set at 37 °C.

### Effect of Mg^2+^ concentration in RNA polymerase reaction

**Fig. 4:**
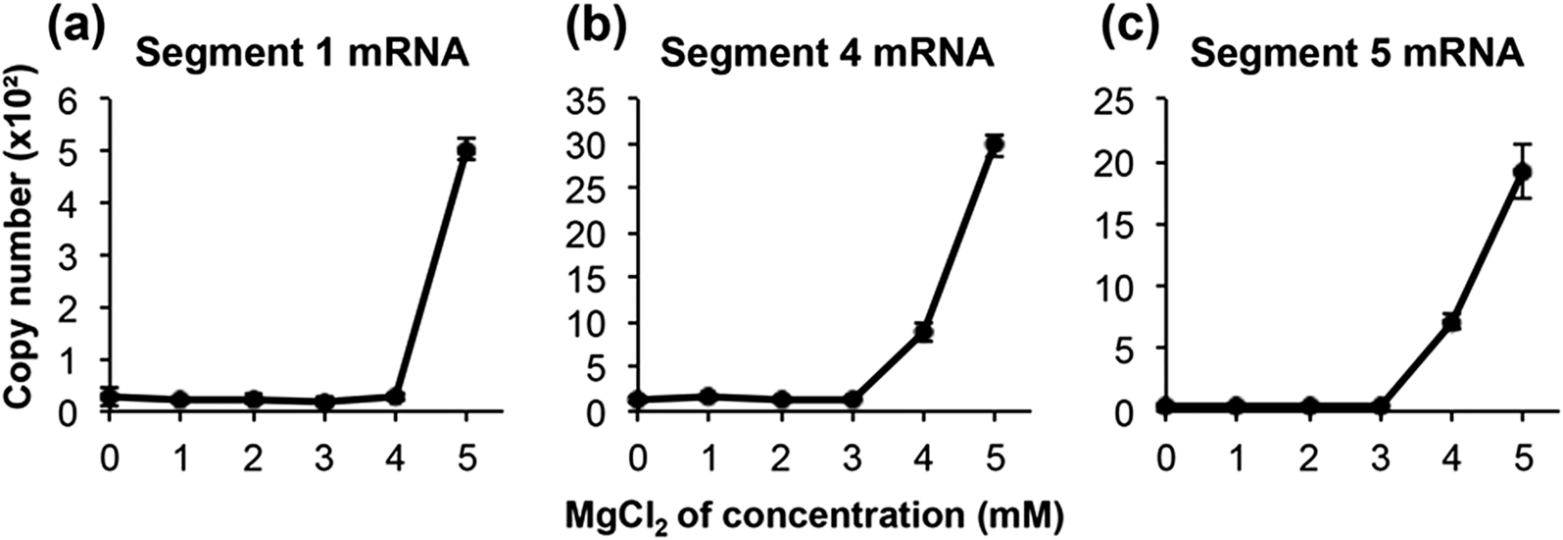
Effect of Mg^2+^ concentration in the RNA polymerase reaction on influenza virus mRNA synthesis. The RNA polymerase reaction for influenza virus was performed under regular reaction conditions in 0, 1, 2, 3, 4, and 5 mM of MgCl_2_. The RNA polymerase reaction mixture was incubated for 30 min at 37 °C. **a** Segment 1 mRNA, **b** segment 4 mRNA, and **c** segment 5 mRNA. Data are presented as mean ± standard deviation (n = 3)

Generally, the polymerase reaction changes depending on the Mg^2+^ concentration in the reaction solution. In segment 1, almost no mRNA was produced up to 4 mM MgCl_2_, but mRNA production increased rapidly at 5 mM MgCl_2_ (Fig. 4a). Segments 4 and 5 showed concentration-dependent production of mRNA at 3 mM to 5 mM of MgCl_2_ (Fig. 4b, c). CaCl_2_ did not affect RdRp activity (data not shown). The reaction solution containing 5 mM MgCl_2_ synthesized 17.8- (Fig. 4a), 23.5- (Fig. 4b), and 55.8-fold (Fig. 4c) more mRNA in segments 1, 4, and 5, respectively, than in the reaction solution without MgCl_2_. From these results, the MgCl_2_ concentration in the RdRp reaction solution was fixed at 5 mM.

### Effect of ApG

**Fig. 5:**
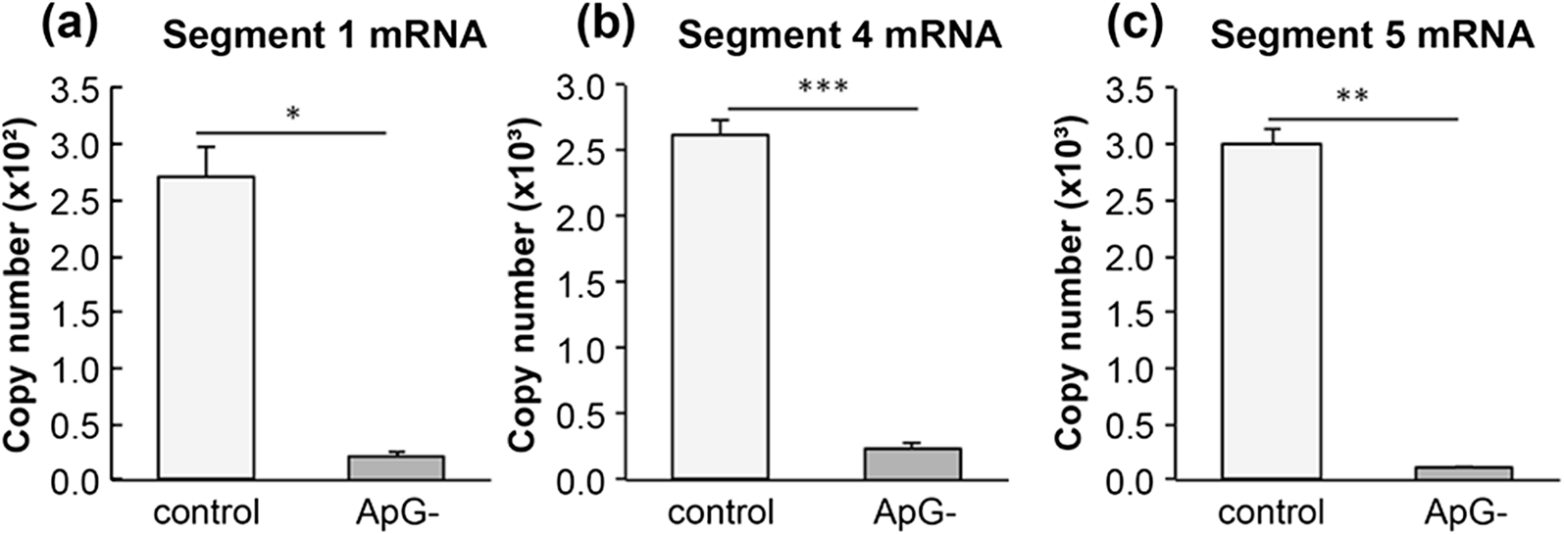
Effect of ApG on influenza virus mRNA synthesis. The RNA polymerase reaction mixture containing 5 mM of MgCl_2_ was incubated for 30 min at 37 °C. The reduction of each mRNA synthesis, **a** segment 1 mRNA, **b** segment 4 mRNA, and **c** segment 5 mRNA, was analyzed with the RNA polymerase reaction without the specific dinucleotide primer ApG. Date are presented as mean ± SD (n = 3). *, *p* < 0.05; **, *p* < 0.001; ***, *p* < 0.0001

The addition of dinucleotide ApG has been reported to significantly accelerate influenza virus mRNA synthesis in the RdRp reaction [12, 17]. Due to the lack of ApG, an initiator of mRNA synthesis, in the RdRp reaction solution, the amount of mRNA synthesis in segments 1, 4, and 5 was markedly reduced to 7.9%, 9.2%, and 3.8%, respectively (Fig. 5a–c).

### The inhibitory effect of the RNA polymerase inhibitor ribavirin triphosphate (RTP)

**Fig. 6:**
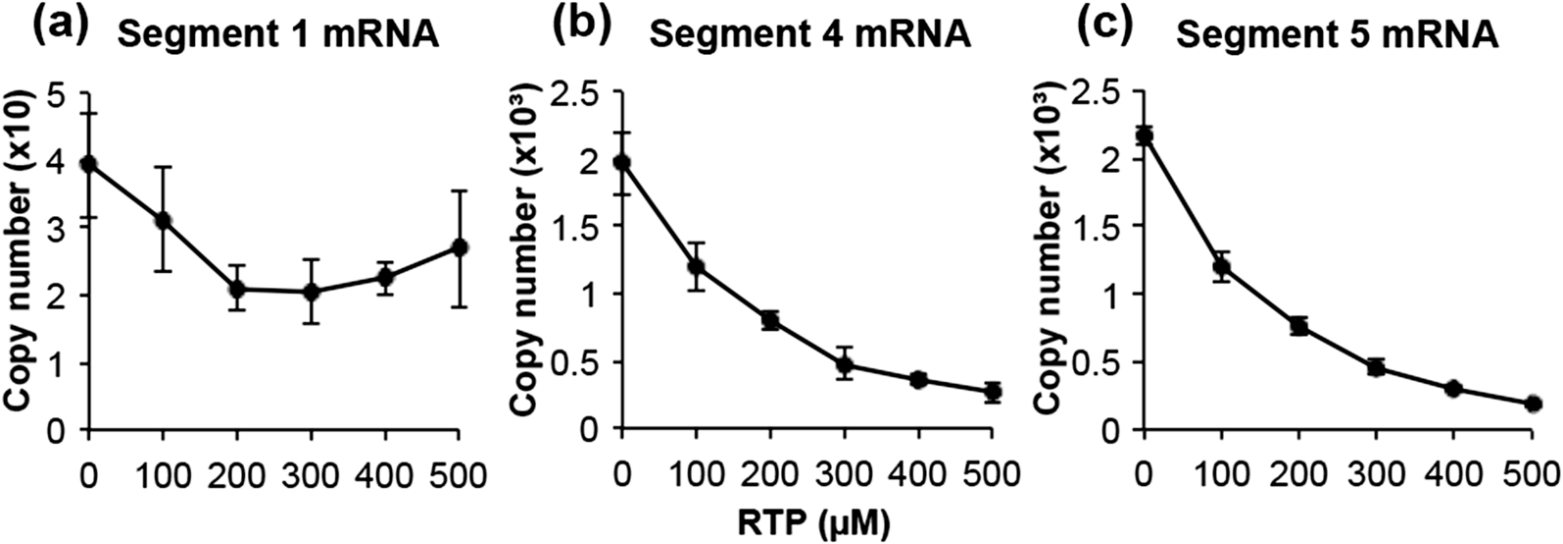
The inhibitory effect of the RNA polymerase inhibitor ribavirin triphosphate (RTP) on influenza mRNA synthesis. The RNA polymerase reaction mixture containing 5 mM of MgCl_2_ was incubated for 30 min at 37 °C. **a** Segment 1 mRNA, **b** segment 4 mRNA, and **c** segment 5 mRNA. Various concentrations of RTP (0^_^500 µM) were added to the polymerase reaction solution. Since RTP is used as a GTP inhibitor, the GTP concentration in this experiment was set to 5 μM. Date are presented as mean ± SD (n = 3)

To investigate whether the method developed in this study to determine RdRp activity might be useful to evaluate RdRp inhibitory compounds, the inhibitory effect of ribavirin triphosphate (RTP), which has been reported to inhibit influenza virus RdRp, was analyzed. In segment 1, 200 µM and 300 µM of RTP inhibited mRNA synthesis by 46.4% and 47.9%, respectively, compared to that of the control (Fig. 6a). In segments 4 and 5, mRNA production was inhibited in a concentration-dependent manner by RTP addition. At 500 µM of RTP in the reaction solution, mRNA production was inhibited in segments 4 and 5 by 86.7% and 91.5%, respectively. This inhibitory effect of RTP in influenza virus RdRp activity did not differ from those previously reported [18, 19] (Fig. 6b, c).

## Discussion

We have established a novel method for measuring the RdRp activity of influenza viruses using RT-PCR. In current methods [17, 20], the amount of mRNA synthesized is quantified using ^32^P-labeled nucleoside triphosphates (GTP or UTP). Therefore, only the total number of eight-segment mRNAs is calculated. However, in the method we developed, the mRNA of each segment can be analyzed individually, and the copy number of the mRNA produced can be quantified through RT-PCR.

In this study, mRNAs of segments 1, 4, and 5 were examined, but by designing the primer using the primer design reported by Kawakami et al. [16] as reference, the amount of cRNAs and vRNAs of each of the eight segments present in influenza virus might be also quantified. As described in the result section, the cRNA and mRNA sequences (positive-sense single-strand) and negative-sense single-strand vRNA sequences are complementary. It is possible to quantify the vRNA of each segment by using a tagged RT primer that specifically binds to the vRNA. The amount of cRNA synthesis can also be measured by using RT primers in which a tag (a sequence different from the tag sequence for mRNA or vRNA) is added to the 3’ end sequences transcribed from vRNA without a poly A tail.

The amount of purified RdRp should be evaluated to avoid errors between experiments. We quantified the amount of vRNA in segments 1, 4, and 5 present in RdRp by real-time qPCR using primers designed as well as mRNA (Additional file 1: Fig. S3; Additional file 2: Table S1). The amount of vRNA present in the purified RdRp was almost the same between these segments. This result indicates that the amount of vRNA in any segment present in RdRp can be used as a reference.

In this study, we attempted to quantify the mRNA levels of HA (segment 4) and NP (segment 5), which are highly expressed among the constituent proteins of influenza virus, and of PB2 (segment 1), which is one of the essential components of RdRp. Kawakami et al. had quantified segments 5 and 6 mRNA in MDCK cells infected with influenza virus (A/WSN/33 strain) [16]. They showed that the expression of segment 5 mRNA was significantly higher than that of segment 6 mRNA [16]. Phan et al. analyzed the expression of mRNA, cRNA, and vRNA of all eight segments in infected cells using RNA sequencing [21]. They demonstrated that the mRNA levels of PB2, PB1, and PA were lower than those of the other five segments at any given time after infection [21]. In our results, the mRNA level of PB2 (segment 1) was lower than that of HA (segment 4) and NP (segment 5). (Figs. 5 and 6). This result indicates that the expression of mRNA may differ between segments, even in in vitro experiments. In addition, in the RTP experiment (Fig. 6), the amount of GTP added to the RdRp reaction solution was reduced, as previously reported [18, 19]. Although the low GTP content in the RdRp reaction solution may have strongly affected the transcription of segment 1 mRNA, the GTP content in each segment mRNA sequence without cap structure was not different (GTP content segment 1, 25.5%; segment 4, 23.2%; segment 5, 26.7%). The reason behind no significant difference in the amount of mRNA between the three segments (Figs. 2 and 3) is unclear. To evaluate the mRNA transcription activity of RdRp using the method developed in this study, it is necessary to evaluate the mRNA transcription levels of multiple segments.

In this study, the conditions for the RdRp reaction were same for the three segments. While measuring the other segments, it is possible to work under the same RdRp reaction conditions as in this study. However, as mentioned above, the transcription amount of each segment may be different. Further research is necessary to determine the optimal conditions for the RdRp reaction for each segment. It is also possible to improve the specificity of primer binding by considering the optimization of the RT reaction and real-time qPCR conditions for each segment.

Viral particles were purified from the culture supernatants of infected cells. Although ultracentrifugation is commonly used to prepare influenza virus polymerase solutions, the method is time consuming and unsuitable for screening. In this experiment, we collected influenza viral particles using magnetic beads. Sakudo et al. have shown through immunochromatography that these beads can efficiently capture influenza viruses in cell culture media [14]. Using this method, it is possible to collect viral particles more easily and quickly than that using ultracentrifugation; therefore, viral particles with high RdRp enzyme activity can be collected. However, as these beads may adsorb various viruses through electrostatic interactions, they are not specific for influenza virus, leading to contamination with other components from the cell culture medium. In our case, the virus polymerase activity was not affected by contamination with the culture medium.

The reaction time for polymerase activity was the same as previously reported [22]. The optimum temperature for this method was 37 °C (Fig. 3), but some previous studies have reported the use of 30 °C [9, 15, 23, 24] Even though studies have reported the use of 37 °C [25, 26], it is unclear why 37 °C was optimum for our method.

Regarding the concentration of Mg in the RNA synthesis reaction buffer, the polymerase activity increased (Fig. 4) at Mg concentrations similar to those previously reported [9, 13, 17]. However, the amplification of each segment increased sharply at 3 or 4 mM MgCl_2_ and showed little increase at concentrations below 3 mM (Fig. 4). Zhang et al. showed a similar rapid increase, although the border concentrations were slightly different [27].

The presence of ApG, a specific dinucleotide primer, increased mRNA production by approximately tenfold in each segment, but mRNA was synthesized to a certain extent even in its absence (Fig. 5).

RTP inhibits RdRp because it is mistakenly taken up during mRNA synthesis as it is similar in structure to GTP and stops mRNA synthesis [18, 19]. Initially, when the GTP concentration was the same as that of ATP, CTP, and UTP, 100 µM RTP had no inhibitory effect, and 500 µM RTP only inhibited segment 1 by 57.1% and segment 4 by 47.1% (data not shown). Therefore, in this experiment we lowered GTP concentration to show the inhibitory effect of RTP. The inhibitory effect of 100 µM RTP was observed resulting in 20.8% inhibition for segment 1, 39.2%, for segment 4, and 44.5% for segment 5 (Fig. 6). In segments 4 and 5, there was no effect of the GTP concentration on mRNA production, and mRNA production decreased depending on the RTP concentration (Fig. 6b, c). However, in segment 1, mRNA production was significantly reduced by the dilution of GTP, even without RTP. Therefore, the concentration-dependent inhibition of RTP could not be confirmed in segment 1 (Fig. 6a). The concentration of GTP is considered important for the synthesis of segment 1 mRNA.

Since the amount of mRNA synthesis was reduced in the absence of ApG or magnesium, and the inhibition of synthesis by RTP was confirmed, this experimental method proved useful at evaluating the activity of RdRp of influenza viruses.

In the analysis of influenza RdRp activity using the dual-luciferase reporter assay, the 5’ and 3' untranslated regions of vRNA (the region recognized and bound by viral polymerase) were inserted into the luciferase reporter system to detect and quantify RdRp activity [28–32]. The advantage of the dual-luciferase reporter assay is that it is useful for high-throughput screening of many compounds. To apply the method developed in this study to high-throughput screening, further ingenuity is required in the process between the RdRp reaction and real-time PCR. However, as the dual-luciferase reporter assay is a method for evaluating intracellular RdRp activity, it may be affected by various intracellular factors. The method developed in this study is an in vitro RdRp reaction; therefore, it is possible to evaluate the direct effect on RdRp. In addition, the dual-luciferase reporter assay requires a luminometer; however, the method developed in this study involves a more commonly used real-time qPCR measuring device.

## Conclusions

This novel method for measuring influenza virus polymerase activity will further promote research to identify compounds that inhibit viral mRNA synthesis.

## Supplementary Information

**Additional file 1: Figure S1.** Graphical representation of the process for extracting RdRp and measuring the copy number of mRNA.** Figure S2**. RdRp component proteins in purified RdRp.** Figure S3**. The vRNA copy numbers of segments 1, 4, and 5 in purified RdRp.

**Additional file 2: Table S1.** Primer sequences for quantitative real-time PCR.

## Data Availability

The datasets analyzed during the current study are available from the corresponding author on reasonable request.

## Abbreviations

HA: Hemagglutinin
MOI: Multiplicity of infection
NP: Nucleoprotein
PA: Acidic polymerase protein
PB1: Polymerase basic protein 1
PB2: Polymerase basic protein 2
qPCR: Quantitative PCR
RdRp: RNA-dependent RNA polymerase
RTP: Ribavirin triphosphate
RNP: Ribonucleoprotein
RT-PCR: Reverse transcription polymerase chain reaction
vRNPs: Viral ribonucleoprotein particles
