# Supplementary material for: Development of a method for evaluating the mRNA transcription activity of influenza virus RNA-dependent RNA polymerase through real-time reverse transcription polymerase chain reaction
Source: Virol J. 2021 Aug 28;18:177. doi: 10.1186/s12985-021-01644-7 (PMC8401337; doi:10.1186/s12985-021-01644-7)
Supplement: Supplementary file 1 — Additional file 1: Figure S1. Graphical representation of the process for extracting RdRp and measuring the copy number of mRNA. Figure S2. RdRp component proteins in purified RdRp. Figure S3. The vRNA copy numbers of segments 1, 4, and 5 in purified RdRp. [file 12985_2021_1644_MOESM1_ESM.pdf]

Additional file 1

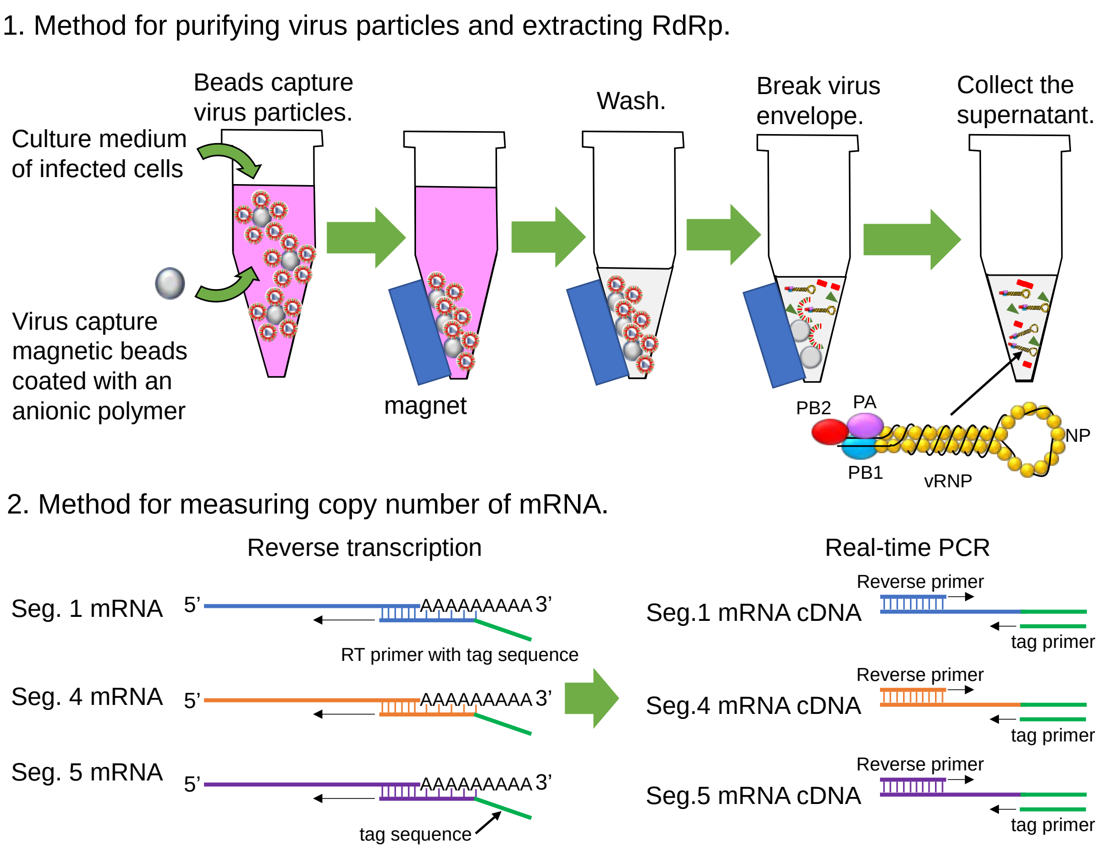

Figure S1 Graphical representation of the process for extracting RdRp and measuring the copy number of mRNA.

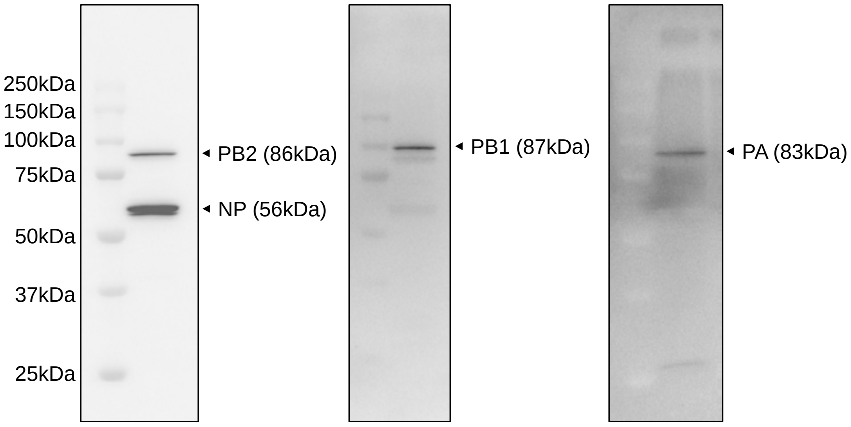

Figure S2 RdRp component proteins in purified RdRp

The purified RdRp solution was subjected to sodium dodecyl sulfate-polyacrylamide gel electrophoresis. RdRp-competent proteins were analyzed by immunoblotting using the following antibodies against each component: anti-PB2 polyclonal antibody (GTX125926, dilution 1/5000; GeneTex, Irvine, CA, USA), anti-PB1 polyclonal antibody (GTX125923, dilution 1/2000; GeneTex, Irvine, CA, USA), anti-PA polyclonal antibody (GTX125932, dilution 1/5000; GeneTex, Irvine, CA, USA), anti-NP polyclonal antibody (GTX125989, dilution 1/10000; GeneTex, Irvine, CA, USA).

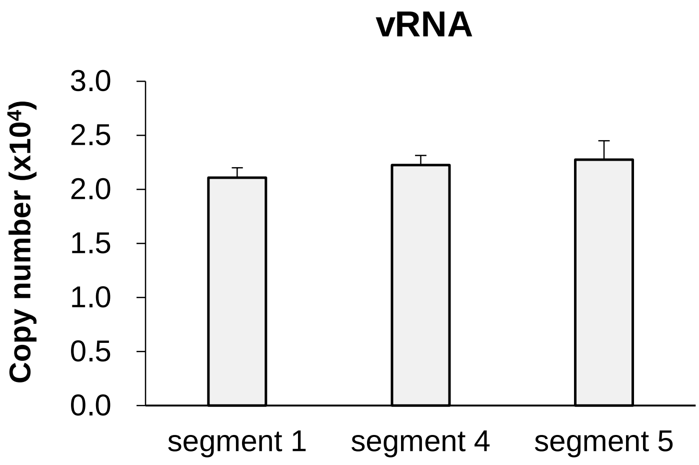

Figure S3 The vRNA copy numbers of segments 1, 4, and 5 in purified RdRp

The vRNA copy numbers of segments 1 (PB2), 4 (HA), and 5 (NP) in purified RdRp were measured using qRT-PCR. vRNA-specific RT primers (Additional file 4: Supplementary Table 1) for segments 1, 4, and 5 of the PR8 strain were designed based on a previous report [16] with some modifications. To prepare cDNA from purified RdRp solution, each tagged-specific RT primer and the ReverTra Ace qPCR RT Kit (TOYOBO, Osaka, Japan) were mixed as follows: 5 μL of a mixture containing 4 μL of the purified RdRp solution and 1 μL of the 5 pmol tagged-specific RT primer. The mixture was heated at 65°C for 10 min, immediately cooled on ice for 5 min, and then heated again at 60°C for 5 min. 5X RT buffer (containing reaction buffer, $MgCl_2$, and dNTPs), RT enzyme mix (containing RT enzyme and RNase inhibitor), and nuclease-free water were added to the purified RdRp solution and primer mixture to a total reaction volume of 10 μL. Real-time quantitative PCR (qPCR) was performed on the ABI PRISM 7500 Fast Real-time PCR system using the Thunderbird SYBR qPCR mix (TOYOBO) and the primer sets for each segment vRNA (Additional file 4: Supplementary Table 1). Each 20 μL of the PCR mixture contained 2 μL of a ten-fold dilution of the cDNA and 0.3 μM of forward and reverse primers. The amplification conditions were 95°C for 1 min, 40 cycles at 95°C for 15 s, 60°C for 30 s, and 72°C for 35 s. Copy numbers were estimated from a calibration curve obtained using serial 10-fold dilutions of the quantified standard sample as the template. Standard samples were generated by inserting the respective amplification range sequences into the pEGFP-N1 plasmid.
