# Supplementary material for: Development of a method for evaluating the mRNA transcription activity of influenza virus RNA-dependent RNA polymerase through real-time reverse transcription polymerase chain reaction
Source: Virol J. 2021 Aug 28;18:177. doi: 10.1186/s12985-021-01644-7 (PMC8401337; doi:10.1186/s12985-021-01644-7)
Supplement: Supplementary file 2 — Additional file 2: Table S1. Primer sequences for quantitative real-time PCR. [file 12985_2021_1644_MOESM2_ESM.docx]

**Additional file 2**

**Table S1 Primer sequences for quantitative real-time PCR**

| Target | Purpose | Prime name | sequences (5’ to 3’) |
| --- | --- | --- | --- |
| Segment 1 vRNA | Reverse transcription | PR8 seg1 vRNA tag | GGCCGTCATGGTGGCGAATGGTGCTTACGGGCAATCTTC |
|  | Realtime PCR | vRNAtag | GGCCGTCATGGTGGCGAAT |
|  |  | PR8 seg1 vRNA Re | TGTTCGTCTCTCCCACTCACTATC |
| Segment 4 vRNA | Reverse transcription | PR8 seg4 vRNA tag | GGCCGTCATGGTGGCGAATTTGCTAAAACCCGGAGACAC |
|  | Realtime PCR | vRNAtag | GGCCGTCATGGTGGCGAAT |
|  |  | PR8 seg4 vRNA Re | CCTGACGTATTTTGGGCACT |
| Segment 5 vRNA | Reverse transcription | PR8 seg5 vRNA tag | GGCCGTCATGGTGGCGAATGAATGGACGAAAAACAAGAATTGC |
|  | Realtime PCR | vRNAtag | GGCCGTCATGGTGGCGAAT |
|  |  | PR8 seg5 vRNA Re | CTCAATATGAGTGCAGACCGTGCT |
